# Critical roles of serotonin-oxytocin interaction during the neonatal period in social behavior in *15q dup* mice with autistic traits

**DOI:** 10.1038/s41598-018-32042-9

**Published:** 2018-09-12

**Authors:** Masatoshi Nagano, Toru Takumi, Hidenori Suzuki

**Affiliations:** 1 Department of Pharmacology, Graduate School of Medicine, Nippon Medical School, 1-1-5 Sendagi, Bunkyo-ku, Tokyo, Japan; 2 RIKEN Brain Science Institute, Wako, Saitama 351-0198 Japan; 3 Core Research for Evolutional Science and Technology (CREST), Japan Science and Technology Agency, Tokyo, Japan

## Abstract

Disturbance of neurotransmitters and neuromodulators is thought to underlie the pathophysiology of autism spectrum disorder (ASD). Studies of *15q dup* mouse models of ASD with human 15q11–13 duplication have revealed that restoring serotonin (5-HT) levels can partially reverse ASD-related symptoms in adults. However, it remains unclear how serotonin contributes to the behavioral symptoms of ASD. In contrast, oxytocin (OXT) has been found to involve social and affiliative behaviors. In this study, we examined whether serotonin-OXT interaction during the early postnatal period plays a critical role in the restoration of social abnormality in *15q dup* mice. OXT or the 5-HT_1A_ receptor agonist 8OH-DPAT treatment from postnatal day 7 (PD7) to PD21 ameliorated social abnormality in the three-chamber social interaction test in adult *15q dup* mice. The effect of 8OH-DPAT was inhibited by blockade of OXT receptors in *15q dup* mice. Thus, serotonin-OXT interaction via 5-HT_1A_ receptors plays a critical role in the normal development of social behavior in *15q dup* mice. Therefore, targeting serotonin-OXT interaction may provide a novel therapeutic strategy for treatment of ASD.

## Introduction

Autism spectrum disorder (ASD) is a neurodevelopmental disorder defined by deficits in social communication and interaction, as well as restricted and repetitive patterns of behavior, interests, or activities (DSM-5). Although various hypotheses have been proposed regarding the pathophysiology of ASD, it has been confirmed that ASD has a strong genetic basis. Copy number variation (CNV) is a significant risk factor for ASD. Using a chromosome engineering technique, we successfully generated a mouse line with duplication of chromosome 7 corresponding to human chromosome 15q11–13^1^, which is one of the CNV loci most frequently identified in cytogenetic abnormalities of ASD^2,3^. Paternal duplication (*15q dup, patDp/*+ in ref.^1^) mice exhibit several abnormal behaviors frequently observed in ASD, such as impaired social interaction and communication, behavioral inflexibility and anxiety-like behaviors^1^. Therefore, *15q dup* mice retain part of the clinical and genetic features of ASD, thus providing an appropriate model for investigating the pathophysiology and therapeutic intervention of ASD. *15q dup* mice also exhibit low serotonin levels in the brain during development^4^. Interestingly, these mice show reversibility of a subset of ASD-related symptoms in adults by the restoration of normal serotonin levels during postnatal development^5^. Although these findings suggest that serotonin may have therapeutic potential for discrete ASD symptoms, the detailed mechanisms of serotonergic modulation remain unclear.

Accumulating evidence indicates that oxytocin (OXT) plays a crucial role in social and affiliative behaviors. Deletion of OXT or the OXT receptor gene results in deficits in social behavior in mice^6–9^. Moreover, single-dose or continuous treatment with OXT improves low sociability in ASD model animals^10–15^ and patients with ASD^16–18^. Therefore, OXT is considered a promising therapeutic candidate for ASD. OXT is released in the brain and acts as a hormone or neuromodulator on a variety of neurotransmitter systems including GABA^19,20^, dopamine^21^ and serotonin^22–26^. Among these mechanisms, the interaction between OXT and serotonin is of particular interest, because both molecules are involved in the control of social behavior^27,28^. OXT receptors are located on serotonergic cells^18,20^, while serotonin (5-HT) receptors are located on OXT neurons^29^. Further, OXT-induced 5-HT release in the nucleus accumbens is reported to support social reward^23^. Considering the reversibility of ASD symptoms using a selective serotonin reuptake inhibitor (SSRI) in our previous study^5^, the serotonin-OXT interaction may underlie the reversible effect of serotonin restoration on social behavior. Therefore, in the current study, we examined whether the OXT system is involved in the effects of serotonergic intervention in *15 dup* mice during the early postnatal period.

## Results

### OXT treatment from PD7 to PD21 ameliorates social abnormality in adult *15q dup* mice

Because a similar early intervention study found that the SSRI fluoxetine (FLX) was effective^5^, we examined whether early postnatal OXT treatment ameliorated the abnormal social interaction observed in adult *15q dup* mice. To this end, we treated mice with OXT from postnatal day (PD) 7 to PD21 and examined their behavior at 8–10 weeks of age.

**Figure 1:**
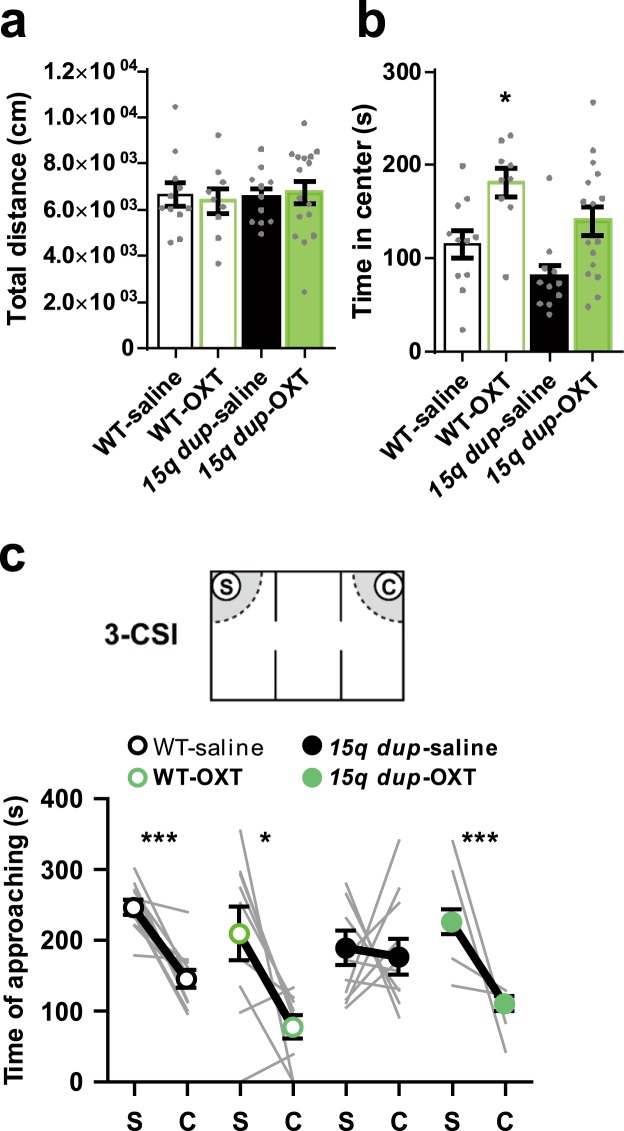
Effects of postnatal OXT treatment on behaviors of adult mice. (**a**) Total distance in OF test. OXT treatment did not affect the total distance traveled in the OF in either WT or *15q dup* mice. (**b**) Time spent in the center area in OF test. Significant drug effect was observed for the time spent in the center (two-way ANOVA; drug: *F*_1,43_ = 16.76, *p* < 0.01). The time spent in the center was significantly increased in WT-OXT mice (Dunnett’s test; *p* = 0.017). (**c**) Approaching time to the stranger cage (S) and the empty cage (C) in the 3-CSI test. There was no significant difference between the time spent around the stranger mouse and the empty cage in *15q dup*-saline mice (WT-saline: t_10_ = 5.980, *p* < 0.001, Cohen’s *d* = 2.65; WT-OXT: t_8_ = 3.165, *p* = 0.013, Cohen’s *d* = 1.48; *15q dup*-saline: t_10_ = 0.272, *p* = 0.791, Cohen’s *d* = 0.15; *15q dup*-OXT: t_15_ = 4.858, *p* < 0.001, Cohen’s *d* = 2.02; paired t-test). The numbers of mice tested and their litters (mice/litters) in each group were as follows. WT-saline (11/4), WT-OXT (9/4), *15q dup*-saline (11/6), *15q dup*-OXT (16/5). Data represent the mean ± SEM. *p < 0.05, ***p < 0.001.

In the open field (OF) test, the OXT treatment did not affect the total distance traveled in the OF in either wild type (WT) or *15q dup* mice (Fig. 1a; two-way ANOVA; interaction: *F*_1,43_ = 0.238, *p* = 0.628; drug: *F*_1,43_ > 0.001, *p* = 0.981; genotype: *F*_1,43_ = 0.066, *p* = 0.798; Dunnett’s test: WT-saline, 6,650 ± 508 cm vs. WT-OXT, 6,390 ± 540 cm, *p* = 0.974: WT-saline vs. *15q dup*-saline, 6,530 ± 351 cm, *p* = 0.997: WT-saline vs. *15q dup*-OXT, 6,770 ± 488 cm, *p* = 0.996). As for the time spent in the center, a significant drug effect was observed. WT and *15q dup* mice who received OXT treatment spent more time in the center than those with the saline treatment, respectively. On the other hand, there were no significant differences between WT mice received saline and *15q dup* mice treated with OXT (Fig. 1b; two-way ANOVA; interaction: *F*_1,43_ = 0.053, *p* = 0.819; drug: *F*_1,43_ = 16.76, *p* < 0.01; genotype: *F*_1,43_ = 5.87, *p* = 0.020; Dunnett’s test: WT-saline, 115 ± 14.9 s vs. WT-OXT, 181 ± 15.2 s, *p* = 0.017: WT-saline vs. *15q dup*-saline, 80.9 ± 12.0 s, *p* = 0.301: WT-saline vs. *15q dup*-OXT, 140 ± 15.5 s, *p* = 0.448).

In the three-chamber social interaction (3-CSI) test, there was no difference between the time spent in the areas around the cage with the stranger mouse and in the areas around the empty cage in *15q dup* mice (Fig. 1c; stranger cage, 188 ± 24.0 s vs. empty cage, 176 ± 25.0 s, t_10_ = 0.272, *p* = 0.791, Cohen’s *d* = 0.15), as we reported in previous studies^1,5^. In contrast, the *15q dup* mice neonatally treated with OXT spent significantly more time around the cage with the stranger mouse than around the empty cage (stranger cage, 226 ± 17.1 s vs. empty cage, 110 ± 10.9 s, t_15_ = 4.858, *p* < 0.001, Cohen’s *d* = 2.02), as did the saline-treated WT mice (stranger cage, 246 ± 10.3 s vs. empty cage, 145 ± 12.4 s, t_10_ = 5.980, *p* < 0.001, Cohen’s *d* = 2.65). In contrast, neonatal OXT treatment did not affect the tendency to spend more time around the stranger cage in WT mice (stranger cage, 209 ± 38.2 s vs. empty cage, 77.0 ± 17.0 s, t_8_ = 3.165, *p* = 0.013, Cohen’s *d* = 1.48).

### 5-HT_1A_ receptor agonist treatment from PD7 to PD21 ameliorates the social abnormality in adult *15q dup* mice

We next examined the mechanisms underlying the restoration of social behavior induced by FLX or OXT in *15q dup* mice. Because 5-HT_1A_ receptor agonists reportedly elevate the plasma OXT levels in rodents^28,29^, we focused on 5-HT_1A_ receptor-mediated effects. Therefore, we treated mice with 8OH-DPAT, a 5-HT_1A_ receptor agonist, from PD7 to PD21, and examined their behavior in adulthood.

**Figure 2:**
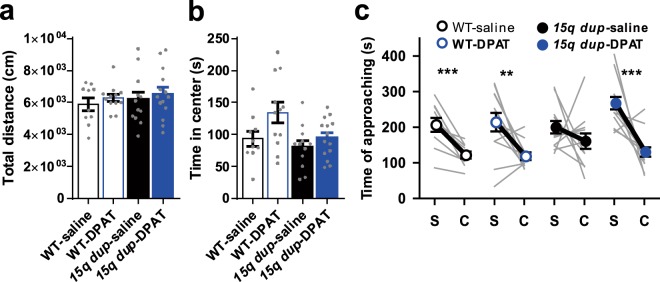
Effects of postnatal 8OH-DPAT treatment on behaviors of adult mice. (**a**) Total distance in OF test. 8OH-DPAT treatment did not affect the total distance traveled in the OF in either WT or *15q dup* mice. (**b**) Time spent in the center area in OF test. Significant drug effect was observed for the time spent in the center (two-way ANOVA; drug: *F*_1,44_ = 5.339, *p* = 0.026), while there were no significant differences among groups. (**c**) Approaching time to the stranger cage (S) and the empty cage (C) in 3-CSI test. There was no significant difference between the time spent around the stranger mouse and the empty cage only in *15q dup*-saline mice (WT-saline: t_9_ = 5.028, *p* < 0.001, Cohen’s *d* = 1.75; WT-DPAT: t_11_ = 3.257, *p* = 0.008, Cohen’s *d* = 1.37; *15q dup*-saline: t_11_ = 1.080, *p* = 0.303, Cohen’s *d* = 0.56; *15q dup*-DPAT: t_13_ = 4.993, *p* < 0.001, Cohen’s *d* = 2.40; paired t-test). The numbers of mice tested and their litters (mice/litters) in each group are as follows. WT-saline (10/4), WT-DPAT (12/6), *15q dup*-saline (12/6), *15q dup*-DPAT (14/6). Data represent the mean ± SEM. **p < 0.01, ***p < 0.001.

In the OF test, the total distance traveled in the OF showed no difference among WT and *15q dup* mice treated with 8OH-DPAT or saline (Fig. 2a; two-way ANOVA; interaction: *F*_1,44_ = 0.005, *p* = 0.944; drug: *F*_1,44_ = 0.892, *p* = 0.350; genotype: *F*_1,44_ = 0.457, *p* = 0.503; Dunnett’s test: WT-saline, 5,890 ± 388 cm vs. WT-DPAT, 6,280 ± 233 cm, *p* = 0.831: WT-saline vs. *15q dup*-saline, 6,180 ± 465 cm, *p* = 0.922: WT-saline vs. *15q dup*-DPAT, 6,520 ± 415 cm, *p* = 0.534). As shown in OXT treatment, a significant drug effect was observed for the time spent in the center in 8OH-DPAT treatment. On the other hand, there were no significant differences among groups (Fig. 2b; two-way ANOVA; interaction: *F*_1,44_ = 1.287, *p* = 0.263; drug: *F*_1,44_ = 5.339, *p* = 0.026; genotype: *F*_1,44_ = 4.888, *p* = 0.032; Dunnett’s test: WT-saline, 93.4 ± 11.9 s vs. WT-DPAT, 134 ± 16.1 s, *p* = 0.062: WT-saline vs. *15q dup*-saline, 80.8 ± 9.7 s, *p* = 0.807: WT-saline vs. *15q dup*-DPAT, 94.6 ± 8.3 s, *p* = 1.000).

In the 3-CSI test, saline*-*treated *15q dup* mice showed no difference in the time spent in the areas around the cage with the stranger mouse and the empty cage (Fig. 2c; *15q dup*-saline, stranger cage, 200 ± 17.9 s vs. empty cage, 161 ± 21.6 s, t_11_ = 1.080, *p* = 0.303, Cohen’s *d* = 0.56), whereas 8OH-DPAT-treated *15q dup* mice spent more time in the area around the cage with the stranger mouse than in the area around the empty cage (*15q dup*-DPAT, stranger cage, 268 ± 17.1 s vs. empty cage, 130 ± 13.2 s, t_13_ = 4.993, *p* < 0.001, Cohen’s *d* = 2.40). Neonatal treatment with 8OH-DPAT did not significantly affect the behavior in WT mice in adulthood (WT-DPAT, stranger cage, 215 ± 26.6 s vs. empty cage, 119 ± 10.5 s, t_11_ = 3.257, *p* = 0.008, Cohen’s *d* = 1.37).

**Figure 3:**
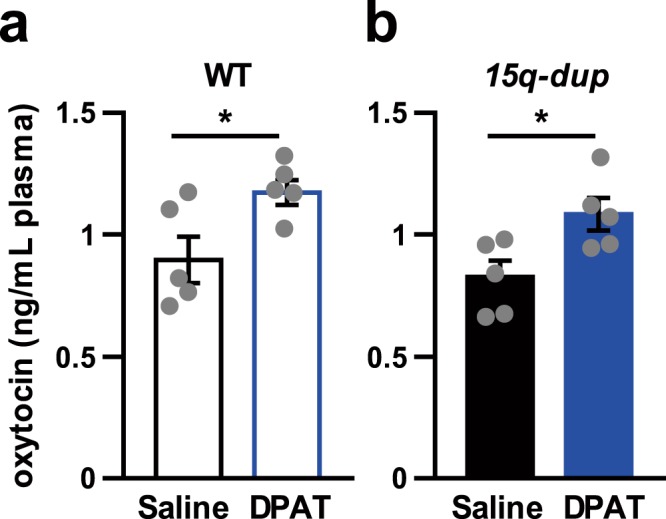
Effects of 8OH-DPAT treatment on plasma OXT levels in 3-week-old mice. (**a**) WT mice (**b**) *15q dup* mice. 8OH-DPAT treatment increased plasma OXT levels both in WT mice (**a**) WT-saline vs. WT-DPAT, t_8_ = 2.606, *p* = 0.031) and *15q dup* mice (**b**) *15q dup*-saline vs. *15q dup*-DPAT, t_8_ = 2.730, *p* = 0.026; Two-tailed Student’s t-tests were applied between treatments in each genotype). The numbers of mice tested and their litters (mice/litters) in each group were as follows. WT-saline (5/3), WT-DPAT (5/3), *15q dup*-saline (5/3), *15q dup*-DPAT (5/3). Data represent the mean ± SEM. *p < 0.05.

We then examined whether 8OH-DPAT affects plasma OXT levels in WT and *15q dup* mice. Ten minutes after subcutaneous injection of 8OH-DPAT at 0.5 mg/kg, the plasma OXT levels were significantly increased in both WT and *15q dup* mice at 3 weeks old (Fig. 3a; WT-saline, 0.895 ± 0.09 ng/mL vs. WT-DPAT, 1.17 ± 0.05 ng/mL, t_8_ = 2.606, *p* = 0.031: Fig. 3b; *15q dup*-saline, 0.824 ± 0.07 ng/mL vs. *15q dup*-DPAT, 1.08 ± 0.07 ng/mL, t_8_ = 2.730, *p* = 0.026). Basal plasma OXT levels were comparable between WT and *15q dup* mice.

### An OXT receptor antagonist treatment from PD7 to PD21 reverses the effect of 8OH-DPAT on social abnormality in adult *15q dup* mice

Finally, we examined whether the effects of neonatal 8OH-DPAT treatment on abnormal social behavior are mediated through OXT receptors. To this end, we administered L-368,899, an OXT receptor antagonist, simultaneously with 8OH-DPAT to mice from PD7 to PD21.

**Figure 4:**
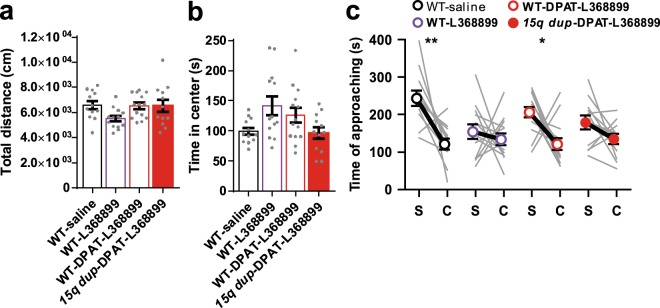
Effects of postnatal L-368,899 treatment on behavior in adult mice. (**a**) Total distance in OF test. There was no significant difference in the total distance traveled among groups (one-way ANOVA: F_3,47_ = 2.488, *p* = 0.072; Dunnett’s test). (**b**) Time spent in the center area in the OF test. There was no significant difference in the center time among groups. (one-way ANOVA: F_3,47_ = 3.418, *p* = 0.025; Steel’s test) (**c**) Time taken to approach to the stranger cage (S) and the empty cage (C) in 3-CSI test. Under the treatment with L-368,899, WT or 8OH-DPAT-treated *15q dup* mice showed no significant difference between the time spent around the stranger mouse and the empty cage (WT-saline: t_11_ = 3.937, *p* = 0.002, Cohen’s *d* = 1.96; WT-L368899: t_12_ = 0.623, *p* = 0.545, Cohen’s *d* = 0.31; WT-DPAT-L368899: t_13_ = 2.948, *p* = 0.011, Cohen’s *d* = 1.50; *15q dup*-DPAT-L368899: t_11_ = 1.805, *p* = 0.098, Cohen’s *d* = 0.80; paired t-test). The numbers of mice tested and their litters (mice/litters) in each group are as follows. WT-saline (12/5), WT-L368899 (13/4), WT-DPAT-L368899 (14/4), *15q dup*-DPAT-L368899 (12/4). Data represent the mean ± SEM. *p < 0.05, **p < 0.01.

In the OF test, L-368,899 treatment did not affect the total distance traveled in the OF in either WT or *15q dup* mice (Fig. 4a; one-way ANOVA: *F*_3,47_ = 2.488, *p* = 0.072; Dunnett’s test: WT-saline, 6,600 ± 329 cm vs. WT-L368899, 5,510 ± 226 cm, *p* = 0.065: WT-saline vs. WT-DPAT-L368899, 6,520 ± 255 cm, *p* = 0.996: WT-saline vs. *15q dup*- DPAT-L368899, 6,510 ± 483 cm, *p* = 0.995). The time spent in the center was unaffected by L-368,899 in either WT or *15q dup* mice, regardless of co-treatment with 8OH-DPAT (Fig. 4b; one-way ANOVA: *F*_3,47_ = 3.418, *p* = 0.025; Steel’s test: WT-saline, 99.6 ± 5.8 s vs. WT-L368899, 142 ± 15.6 s, *p* = 0.25: WT-saline vs. WT-DPAT-L368899, 125 ± 12.4 s, *p* = 0.522: WT-saline vs. *15q dup*-DPAT-L368899, 96.4 ± 9.3 s, *p* = 1).

In the 3-CSI test, L-368,899-treated WT mice spent a similar amount of time around the area of the stranger mouse cage and the empty cage (Fig. 4c; WT-L368899, stranger cage, 154 ± 19.4 s vs. empty cage, 134 ± 15.9 s, t_12_ = 0.623, *p* = 0.545, Cohen’s *d* = 0.31). Treatment with L-368,899 reversed the 8OH-DPAT-induced restoration of social behavior in *15q dup* mice (Fig. 4c; *15q dup*-DPAT-L368899, stranger cage, 179 ± 18.2 s vs. empty cage, 135 ± 13.6 s, t_11_ = 4.993, *p* = 0.098, Cohen’s *d* = 0.80). On the other hand, WT mice treated with both L-368,899 and 8OH-DPAT showed similar behavior to the saline-treated WT mice (Fig. 4c; WT-DPAT-L368899, stranger cage, 206 ± 14.1 s vs. empty cage, 121 ± 16.0 s, t_13_ = 2.948, *p* = 0.011, Cohen’s *d* = 1.50).

## Discussion

The current results revealed that early postnatal OXT treatment ameliorated abnormal social behavior in adult *15q dup* mice with a similar treatment protocol to the method we adopted to successfully restore the abnormal social interaction with FLX in a previous study^5^. Importantly, early postnatal OXT treatment had a long-term benefit on sociability. In addition, treatment with the OXT receptor antagonist L-368,899 from PD7 to PD21 impaired sociability in adulthood in WT mice. Treatment with another OXT receptor antagonist within 24 hours after birth was previously reported to impair sociability in adulthood in female mice^30^. These findings suggest the importance of OXT during the neonatal period for the development of normal social behavior in adulthood. Accordingly, many reports have suggested that impairment of the OXT system affects sociability^6–9^, and OXT is effective for restoration of disturbed sociability in a variety of ASD model animals^10–15^. Clinical studies have also reported that symptoms in patients with ASD are partially improved by OXT applied via nasal spray^16–18^. In the current study, OXT treatment was found to be effective for improving social behavior in *15q dup* mice, as observed in other ASD model animals and patients with ASD. These findings suggest that *15q dup* mice have predictive validity as well as face validity and construct validity as an animal model of ASD.

In the present study, we adopted the subcutaneous injection to deliver precise amount of OXT to immature mice, and observed significant effects of oxytocin on social behaviors compared with the saline. Several studies have shown that intranasal administration of oxytocin increases its concentrations in both brain tissue and plasma^31^ and affects behaviors relating higher brain functions^12^. On the other hand, there also have been several reports showing that peripheral injection of oxytocin increases its concentrations in both brain tissue and plasma with a pharmacokinetic profile different from nasal delivery^31^, and induces some behavioral changes relating higher brain functions^14^, consistent with the present results. Further, because we administered a pharmacological dose of oxytocin (i.e. an excess dose compared with endogenous peptide dose) to postnatal mice, which may have more immature brain-blood barrier than adult mice, oxytocin could more easily penetrate the brain-blood barrier.

The current findings demonstrated that the 5-HT_1A_ receptor agonist 8OH-DPAT administered in the early postnatal period ameliorated the impaired social behavior in adult *15q dup* mice, similar to the effects of treatment with FLX. These results suggest that 5-HT_1A_ receptors may be at least partially involved in the restorative effects of early serotonergic intervention on abnormal sociability in adult *15q dup* mice. 5-HT_1A_ receptor agonists have been reported to elevate the plasma OXT levels in rodents^32,33^. OXT neurons are located in the hypothalamus and express 5-HT_1A_ receptors^29^. In the present study, we confirmed that 8OH-DPAT increased plasma OXT levels both in WT and *15q dup* mice. Further, the OXT receptor antagonist L-368,899 blocked the 8OH-DPAT-induced restoration of social behavior in *15q dup* mice. These lines of evidence suggest that the OXT system acts downstream of the serotonergic systems to facilitate normal development of social behavior. However, we found that L-368,899 blocked normal social behavior in WT mice, but not in WT mice treated with 8OH-DPAT. The reason for the discrepancy in these effects is unclear. Experimental conditions such as dose or specificity of the antagonist may have affected the results. Alternatively, because OXT receptors are expressed on serotonergic cells^22,24^, the OXT system may inversely exert effects on serotonergic neurons. Further, there may be some differences in responsiveness to OXT between WT and *15q dup* mice, including the OXT receptor expression level in the brain. Interestingly, it has been previously reported that functional serotonin-OXT interactions are altered in the brain in patients with ASD^34^.

In conclusion, the current findings indicate that OXT is effective for tr eating impaired sociability in *15q dup* mice, consistent with the effects observed in other ASD model animals with different genetic mutations^10,13–15^. In addition to the OXT system, serotonin-OXT interactions via 5-HT_1A_ receptors play a critical role in the normal development of social behavior. Therefore, targeting serotonin-OXT interactions may provide another novel therapeutic strategy for treatment of ASD.

## Methods

### Animals

We tested male *15q dup* mice^1,5^ and their littermate male wild type (WT) C57BL/6J mice. All mice were kept under constant temperature (22 ± 1 °C) on a regular light/dark cycle (lights on from 06:00 to 20:00 h), with free access to food and water. All experiments were conducted in accordance with the National Institutes of Health Guide for the Care and Use of Laboratory Animals. This study was reviewed by the Institutional Animal Care and Use Committee and approved by the President of the Nippon Medical School (Approval Number: 27-035).

### Drug treatment

(R)-(+)-8-hydroxy-2-(di-*n*-propylamino)tetralin hydrobromide (8OH-DPAT; Sigma, USA), a specific 5-HT_1A_ receptor full agonist, L-368,899 hydrochloride (Tocris, UK), an OXT receptor antagonist, or OXT (Sigma, USA) was dissolved in saline and subcutaneously injected to pups (8OH-DPAT, 0.5 mg/kg; L-368,899, 3 mg/kg; OXT 83 I.U. [0.2–0.26 mg]/kg) every day from PD7 to PD21.

The treatment doses of 8OH-DPAT^35^, L-368,899^36,37^ and OXT^30^ were determined based on previous reports. The treatment duration of each drug was determined based on our previous reports^5^. After weaning at PD21, the mice were divided into groups of two to five mice per cage. Saline was used as a control.

### Measurement of plasma OXT

Three-week-old *15q dup* and wild type (WT) mice were subcutaneously administered with saline or 8OH-DPAT (0.5 mg/kg). Ten minutes later, the mice were decapitated under deep anesthesia with halothane and the blood was collected. The plasma OXT was assayed with an extraction-free enzyme immunoassay kit (Phoenix Pharmaceuticals, Inc., USA).

### Behavioral tests

All behavioral tests were performed between 08:30 and 14:00 h on mice at 8–10 weeks of age (*n* = 9–16 per group). The apparatuses and software used for behavioral analyses were produced by O’Hara & Co. Ltd. (Tokyo, Japan). The OF and 3-CSI tests were conducted as previously described^5^. Briefly, the OF test was conducted prior to the 3-CSI test. Behavior was monitored for 15 min and recorded with a CCD camera connected to a personal computer. The ambulation distance and the time spent in the center of the field (a central square of 30 × 30 cm) were automatically recorded and analyzed. For the 3-CSI test, each subject mouse was acclimated to the test box by free exploration for 5 min before the test. Subsequently, an unfamiliar younger WT C57BL/6J male mouse (stranger mouse) that had no prior contact with the subject mouse was placed in a wire cage at one corner, while the other cage at the opposite corner remained empty. After the subject mouse was placed in the middle chamber to freely move throughout all three chambers, the behavior was recorded for 10 min and the time spent in the interaction zone (an area within 9 cm from the edge of the wire cage) was automatically measured for the approaching time. Because the 3-CSI system used in the present study can automatically calculate the duration of stay time (in each cage) as the approaching time without distinguishing the posture of the mice (facing to the stranger mouse or not), we manually examined the posture of the mice around the stranger cage by placing a video camera near the cage in seven mice and compared the result of the automated system in a preliminary experiment. We found that the time spent interaction zone obtained from the automated system was highly correlated with the time of facing to the stranger mouse manually counted (r = 0.93), confirming examined approaching (stay) time around the stranger cage as a reliable index of social behavior in the system^1,5^. The observer was blinded to the mouse genotypes until all behavioral tests were finished.

### Statistics

All data are expressed as means ± SEM. For OF tests, two-way ANOVA (Figs 1a,b and 2a,b) or one-way ANOVA (Fig. 4a,b) was used. Then, as post hoc analysis, Dunnett’s test (Figs 1a,b, 2a,b and 4a) or Steel’s test (Fig. 4b) was used for comparison of values between individual groups. For 3-CSI tests, a paired t-test was used to compare the time spent in the interaction zone of the cage with the stranger mouse with that of the empty cage within each strain or within each treatment group, following papers^38,39^ by Crawley’s group. Cohen’s *d* values were calculated online (http://www.socscistatistics.com/effectsize/Default3.aspx) to express the effect size for the significant pairwise comparisons (Cohen’s *d* values greater than 0.5 were considered to be medium in strength, while values greater than 0.8 were considered to have a large effect size)^34^. The analyses were performed using GraphPad Prism software (San Diego, USA) and KyPlot software (KyensLab Inc., Tokyo, Japan). For plasma OXT levels, treatment difference was assessed using two-tailed Student’s t-tests in each genotype. *P*-values < 0.05 were considered statistically significant.

## Data Availability

The datasets generated during and/or analyzed during the current study are available from the corresponding author on reasonable request.
